# Prognostic Value of Initial Inflammatory Biomarkers, ECG Findings, and Computed Tomography in the Assessment of Acute Pulmonary Embolism Severity

**DOI:** 10.3390/medicina61101830

**Published:** 2025-10-13

**Authors:** Bojana Uzelac, Vladimir Jakovljević, Vladimir Živković, Jelena Janković, Katarina Lazarević, Danilo Marković, Marija Laban-Lazović, Andrija Jovanović, Marina Đikić, Dušica Gujaničić, Ivana Milićević-Nešić, Sanja Stanković

**Affiliations:** 1 Emergency Center, University Clinical Center of Serbia, 11000 Belgrade, Serbia; dr.andrijajovanovic@gmail.com (A.J.); marina.djikic81@gmail.com (M.Đ.); dusskag@gmail.com (D.G.); milicevic.iv@gmail.com (I.M.-N.); 2 Department of Physiology, Faculty of Medical Sciences, University of Kragujevac, 34000 Kragujevac, Serbia; drvladakgbg@yahoo.com (V.J.); vladimirziv@gmail.com (V.Ž.); 3 Center of Excellence for Redox Balance Research in Cardiovascular and Metabolic Disorders, 34000 Kragujevac, Serbia; 4 Department of Human Pathology, First Moscow State Medical University I.M. Sechenov, 119146 Moscow, Russia; 5 Department of Pharmacology, First Moscow State Medical University I.M. Sechenov, 119146 Moscow, Russia; 6 Clinic for Pulmonology, University Clinical Center of Serbia, 11000 Belgrade, Serbia; jjelena1984@gmail.com (J.J.); marija.labanlazovic@gmail.com (M.L.-L.); 7 Center for Radiology and Magnetic Resonance, University Clinical Center of Serbia, 11000 Belgrade, Serbia; katarinalazarevic76@gmail.com (K.L.); markovic.danilo.md@gmail.com (D.M.); 8 Center for Medical Biochemistry, University Clinical Center of Serbia, 11000 Belgrade, Serbia; sanjast2013@gmail.com; 9 Department of Biochemistry, Faculty of Medical Sciences University of Kragujevac, 34000 Kragujevac, Serbia

**Keywords:** acute pulmonary thromboembolism, Daniel’s ECG score, hemogram-derived ratios

## Abstract

*Background and Objectives:* Acute pulmonary thromboembolism (PTE) is one of the leading causes of cardiovascular mortality. Recent insights into PTE pathophysiology emphasize the complex interplay of multiple mechanisms, particularly the roles of thrombosis and inflammation. *Methods:* This retrospective, single-center observational study included 138 participants: 69 adult patients diagnosed with PTE via computed tomography pulmonary angiography (CTPA) and 69 matched healthy controls. Upon admission, a standard 12-lead electrocardiogram (ECG) was performed, and Daniel’s score was calculated. Peripheral blood samples were collected to assess inflammatory biomarkers and hemogram-derived ratios (SII, NLR, dNLR, NPR, PLR, LMR). CTPA scans were analyzed not only for diagnostic purposes and PTE localization but also for inflammatory changes. PTE severity was classified according to the 2019 ESC guidelines. *Results:* Patients with PTE had significantly higher Daniel’s ECG scores, initial values of inflammatory biomarkers (WBC, neutrophils, IL-6, CRP) and hemogram-derived ratios (SII, NLR, dNLR, NPR) compared to controls. In multivariate analysis, older age (OR = 1.05; *p* = 0.038), higher Daniel’s ECG score (OR = 1.24; *p* < 0.001), and higher dNLR (OR = 1.40; *p* = 0.001) were found as an independent predictors of PTE severity. Ground-glass opacity (GGO) was the most common parenchymal and pleural inflammatory finding relating to CTPA (48.4%), but these findings did not show significant predictive value for PTE severity. *Conclusions:* Daniel’s ECG score and dNLR, both readily available and cost-effective biomarkers demonstrated independent predictive value for assessing PTE severity.

## 1. Introduction

Acute pulmonary thromboembolism (PTE) is the third leading cause of cardiovascular death, following acute myocardial infarction and stroke, with an estimated incidence of 75 to 269 events per 100,000 individuals and approximately 60,000 to 100,000 annual deaths [1,2,3].

The modern concept of PTE pathophysiology is closely linked to endothelial injury, inflammation, hypercoagulability, and hemodynamic disorders. Pro-inflammatory cytokines (IL-1β, IL-6, IL-8) activate tissue factors, exhibit pro-coagulant effects, trigger platelet adhesion and aggregation, promote vasoconstriction, and may induce thrombosis. Sympathetic activation during acute stress affects leukocyte distribution by increasing neutrophil counts and decreasing lymphocyte counts. These mechanisms are reflected in hemogram-derived inflammatory ratios, low-cost markers easily calculated from a routine complete blood count (CBC) [4,5,6,7,8]. Although these ratios have proven useful as markers of inflammatory response in various pathological conditions [6,7,8], only a few studies have investigated their significance in PTE patients [9,10,11,12,13,14,15].

Acute PTE causes a sudden increase in pulmonary vascular resistance, leading to right ventricular (RV) pressure overload and acute dilation. This mechanical stress is reflected in ECG changes indicative of RV strain, such as sinus tachycardia, right bundle branch block (RBBB), ST-segment changes, T-wave inversions, and right axis deviation. Additionally, PTE triggers the release of inflammatory mediators, causing myocardial edema, oxidative stress, and direct injury to the RV myocardium-factors that further compromise hemodynamics, electrical stability, and ECG findings [16,17,18,19].

In 2001, Daniel et al. [20] proposed a scoring system that incorporates several ECG changes predictive of increased pulmonary arterial pressure and adverse clinical outcomes, including death, in patients with PTE. Subsequent investigations confirmed the diagnostic value of this score and expanded it beyond its original components (tachycardia, S1Q3T3 pattern, RBBB/incomplete RBBB, and T-wave inversions in leads V1–V4) to also include ST-segment elevation in lead aVR and atrial fibrillation (AF). All six ECG abnormalities were associated with an increased risk of circulatory shock and 30-day mortality in PTE patients [21,22]. However, despite these findings, Daniel’s ECG score remains underutilized in daily clinical practice.

While computed tomography pulmonary angiography (CTPA) is well established as the gold standard for diagnosing PTE [23], its clinical utility extends beyond diagnosis. CTPA provides detailed visualization of thrombus location, which plays a key role in PTE risk stratification. More centrally located thrombi (in the main or lobar pulmonary arteries) are associated with a higher clot burden and worse clinical outcomes. In addition to thrombotic findings, CTPA can also reveal parenchymal and pleural inflammatory changes such as ground-glass opacities (GGO), consolidation, pleural effusion, and lymphadenopathy. Although systemic inflammatory biomarkers (e.g., WBC, CRP, IL-6) and hemogram-derived inflammatory ratios have been linked to PTE severity and mortality, data on the prognostic significance of inflammatory findings on CTPA remain limited [24,25,26,27,28,29,30].

This study aims to determine whether initial systemic inflammatory biomarkers, hemogram-derived inflammatory ratios, Daniel’s ECG score, or CTPA-detected inflammatory findings can serve as independent predictors of PTE severity.

## 2. Materials and Methods

### 2.1. Study Design and Participants

This retrospective observational study was conducted at the University Clinical Centre of Serbia (UCCS), within the Emergency Center, from March 2023 to February 2024. A total of 138 adults were included: 69 patients with PTE diagnosed via CTPA and 69 age- and sex-matched healthy volunteers without prior comorbidities, who served as controls.

Patients with confirmed PTE were stratified according to the 2019 European Society of Cardiology (ESC) guidelines [23] into four risk categories: low risk (*n* = 17, 24.6%), intermediate-low risk (*n* = 30, 43.5%), intermediate-high risk (*n* = 19, 27.5%), and high risk (*n* = 3, 4.3%). Due to the small number of high-risk patients, the intermediate-high and high-risk groups were combined for statistical analysis.

### 2.2. Laboratory Analysis

At admission, peripheral venous blood samples were collected for standard biochemical analyses and measurement of inflammatory biomarkers, including white blood cell (WBC) count, neutrophils, interleukin-6 (IL-6), and C-reactive protein (CRP). Hemogram-derived inflammatory ratios were calculated as follows [6]:

SII = (platelet count × neutrophil count)/lymphocyte count

NLR = neutrophils/lymphocytes

dNLR = neutrophils/(leukocytes − neutrophils)

PLR = platelets/lymphocytes

NPR = neutrophils/platelets

LMR = lymphocytes/monocytes

### 2.3. Electrocardiography (ECG)

All subjects underwent a standard 12-lead ECG using a Schiller Cardiovit AT-102 G2 device. The components of Daniel’s ECG score [20] were recorded and scored, including: sinus tachycardia, complete or incomplete right bundle branch block (RBBB/iRBBB), T-wave inversion (TWI) in leads V1–V4, and the S1Q3T3 pattern.

### 2.4. Computed Tomography Pulmonary Angiography (CTPA)

CTPA was performed using a 64-row Siemens Somatom Drive CT scanner following a standard protocol: an intravenous injection of 100 mL of contrast medium at a flow rate of 4 mL/s, with lung scanning from the base to the apex in a caudocephalic direction at a tube voltage of 120 kV during an inspiratory breath-hold [29].

Pulmonary embolism was identified as partial intraluminal filling defects or complete occlusion of the pulmonary artery observed on two consecutive CT slices. Thrombus location was classified as main (left or right), lobar, or segmental. Inflammatory parenchymal and pleural findings were defined as follows: ground-glass opacity (an area of increased lung attenuation), consolidation (a dense, homogeneous opacity that obscures vessels and airway walls), pleural effusion (a fluid-density collection in one or both pleural spaces), and lymphadenopathy (mediastinal or hilar lymph nodes larger than 10 mm in short axis) [31].

All CTPA scans were independently reviewed by two radiologists blinded to the clinical and laboratory data. Any discrepancies were resolved by consensus. Seven patients who had already undergone CTPA at another medical facility before referral to the Emergency Center were excluded from the analysis. Therefore, a total of 62 CT scans were evaluated by the radiologists at the Emergency Center.

## 3. Exclusion Criteria

Patients were excluded from the study if they met any of the following criteria: age > 70 years, chronic renal insufficiency (creatinine > 400 µmol/L), morbid obesity (weight > 160 kg, CT machine limit), or death prior to hospital admission.

## 4. Statistical Analysis

Depending on the type of variables and the normality of the distribution, the data description is presented as n (%), arithmetic mean ± standard deviation, or median (min-max). Among the methods for testing statistical hypotheses, we used: *t*-test, Mann–Whitney test, chi-square test, or Fisher’s exact probability test. Ordinal logistic regression was used to model the relationship between the dependent variable (degree of PTE) and potential predictors. Predictors from univariate analyses that were statistically significant at the significance level of 0.1 were included in the multivariate regression models. Statistical hypotheses were tested at a statistical significance level (alpha level) of 0.05. The results are presented tabularly and graphically. All data were processed using the IBM SPSS Statistics 24 software package (SPSS Inc., Chicago, IL, USA).

## 5. Results

A total of 138 subjects were included in the study: 69 patients with PTE diagnosed by CTPA (66.7% male, mean age 54.9 ± 11.8 years) and 69 age- and sex-matched healthy controls (66.7% male, mean age 55.3 ± 11.6 years).

Patients with PTE had significantly higher Daniel’s ECG scores at admission (*p* < 0.001) and significantly elevated initial levels of inflammatory biomarkers, including WBC, neutrophils, IL-6, and CRP (*p* < 0.001). Hemogram-derived inflammatory ratios (SII, NLR, dNLR, NPR) were significantly higher in the PTE group, whereas LMR was significantly lower (*p* < 0.001). No statistically significant difference was observed in PLR levels between groups (*p* = 0.063). Clinical characteristics and laboratory findings are summarized in Table 1.

More than half of the patients, 36 (58.1%), had embolism in at least one of the main pulmonary arteries: 22 (35.5%) in both main branches and 14 (22.6%) in a single main artery. Lobar PTE was identified in 18 patients (29.0%), while segmental PTE was observed in 8 patients (12.9%). Parenchymal and pleural inflammatory changes detected on CTPA included ground-glass opacity (48.4%), pleural effusions (unilateral or bilateral) (32.3%), mediastinal or hilar lymphadenopathy (30.6%), and parenchymal consolidation (29.0%). The distribution of PTE and CTPA inflammatory findings is summarized in Table 2.

The univariate model of ordinal logistic regression, with the degree of PTE as the dependent variable, is shown in Table 3.

**Figure 1:**
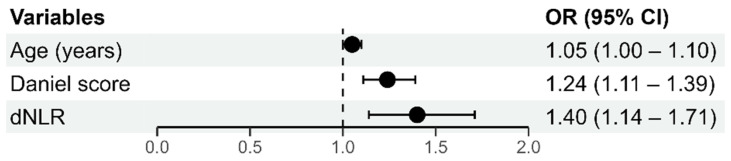
Graphical representation of the multivariate ordinal logistic regression model with the degree of PTE as the dependent variable.

The multivariate model of ordinal logistic regression with the degree of PTE as a dependent variable, is shown in Figure 1. The whole model (with all predictors) was statistically significant (*p* < 0.001) and accounted for 42% of the variation in the dependent variable. Due to multi-collinearity with the dNLR variable, the NLR variable was not included in the multivariate model.

Statistically significant predictors of PTE severity included older age (OR = 1.05; *p* = 0.038), higher Daniel’s ECG score (OR = 1.24; *p* < 0.001), and elevated initial dNLR values (OR = 1.40; *p* = 0.001).

**Figure 2:**
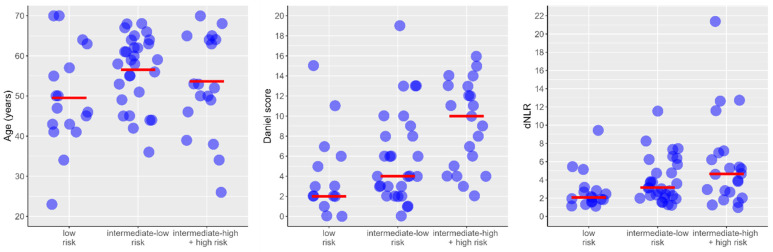
Individual predictor of PTE severity from the multivariate model (represented by blue circles). The red dashed lines represent the mean for age and the median for Daniel score and dNLR values.

The relationship of individual predictors of PTE severity from the multivariable model is shown in Figure 2.

## 6. Discussion

This study is the first to comprehensively assess Daniel’s ECG score, inflammatory hemogram-derived ratios, and CTPA findings in assessing PTE severity. Our results demonstrate that both dNLR and Daniel’s ECG score are statistically significant independent predictors of PTE severity.

Earlier studies found no sex-related difference in total PTE incidence; however, the pattern varies across age groups. Among individuals aged 20–40, women experience PTE at nearly twice the rate of men, whereas after age 60, approximately 25% higher incidence is observed in men [32].

In our study, two-thirds of PTE patients were men (66.7% male and 33.3% female), with a mean age of 54.9 ± 11.8 years. Age was a statistically significant predictor of a higher degree of PTE (OR = 1.05; *p* = 0.038). Each additional year of age increased the likelihood for higher degree of PTE by 5%.

The interplay between inflammation and thrombosis has gained increasing attention, particularly following the COVID-19 pandemic and its virus-induced hypercoagulability [6,33,34]. Afzal et al. first reported a correlation between elevated WBC and neutrophil counts and PTE [35], while Huang C.M. et al. later identified WBC ≥ 11,000/mm^3^ as an independent predictor of 30-day mortality [36]. C-reactive protein (CRP) is well-known to be elevated in deep vein thrombosis (DVT) and PTE [37,38]; however, recent studies remain inconclusive regarding its direct inflammatory role [39], and some have found no predictive value of CRP for PTE severity [9]. Bontekoe et al. [4] reported upregulation of inflammatory cytokines (IL-4, IL-6, IL-8, IL-10, and IL-1β) in PTE patients, and several studies have demonstrated an association between IL-6 levels and PTE mortality [40].

Consistent with previous studies [35,36,37,38,39,40], our results also showed elevated inflammatory biomarkers (WBC, neutrophil counts, CRP, and IL-6) in PTE patients compared with healthy controls. However, these markers did not demonstrate statistically significant predictive value for PTE severity.

Recent research on hemogram-derived ratios has shown a strong correlation with pulmonary embolism. Gok et al. [9] reported elevated SII levels in PTE patients, correlating with PTE severity. Both PLR and NLR were found to be increased in high-risk PTE [10,11,12,13], with NLR recognized as an independent predictor of early mortality [14]. Although dNLR has been identified as an outcome predictor in other conditions such as COVID-19 and malignancy [41,42], it has not yet been investigated in PTE. NPR values have previously been studied in COVID-19 PTE patients [33]. Prior to this paper, only a few studies investigated LMR levels in PTE patients, concluding that lower LMR was an independent predictor of short-term mortality [15].

In our study, inflammatory hemogram-derived ratios, SII, NLR, dNLR, and NPR, were significantly higher in PTE patients (*p* < 0.001), while LMR values were significantly lower. Higher dNLR values were also significantly associated with PTE severity (OR = 1.40; *p* = 0.001).

ECG changes in PTE are well-known to result not only from mechanical right ventricular (RV) dysfunction and overload but are amplified by inflammation-induced myocardial injury and stress responses. Although ECG changes in PTE are often nonspecific, some reflect RV strain. In 2001, Daniel et al. [20] identified several ECG findings in PTE patients (tachycardia, S_1_Q_3_T_3_ pattern, RBBB/iRBBB, and TWI in V1–V4) and integrated them into an ECG scoring system (0–21), with scores above 8 predicting adverse clinical outcomes such as death, shock, or respiratory failure. Although subsequent studies [21,22] confirmed its validity and simplicity, this scoring system has not gained widespread clinical use.

We found that Daniel ECG score was significantly higher in PTE patients than in controls (*p* < 0.001). We also identified the Daniel ECG score as a significant predictor of PTE severity (OR = 1.24; 95% CI: 1.11–1.39; *p* < 0.001). Each one-point increase in the Daniel score raised the likelihood of more severe PTE by 24%, after adjusting for other model variables.

Regarding PTE localization on CTPA, a meta-analysis found that neither total thrombus burden nor the most proximal PTE localization was correlated with all-cause mortality; nevertheless, both were predictive of adverse clinical outcomes [43].

Patients in our study exhibited a substantial embolic burden: more than half (58.1%) had embolism involving at least one main pulmonary artery, lobar PTE was observed in 29.0% of patients, and segmental PTE in 12.9%. However, these variables were not included in the final statistical model for two main reasons. First, involvement of the main pulmonary arteries (one or both) is inherently linked to higher PTE severity, making localization a proxy for the outcome rather than an independent predictor. Therefore, including it would introduce redundancy into the model. Second, PTE localization showed multicollinearity with the Daniel ECG score, already included as a predictor in the model.

Although the role of blood-based inflammatory biomarkers in PTE has been extensively studied, only few studies have examined inflammatory findings on CTPA in this population. Pfeil et al. [24] reported a correlation between wedge-shaped opacities and PTE, while Lee et al. [25] demonstrated a significant correlation between wedge-shaped peripheral consolidation and PTE in children. An animal model of acute PTE concluded that PTE triggers GGO in unobstructed lung regions, most likely due to the redistribution of blood flow. [26] Panjwani et al. [27] reported pleural effusion in 35% of PTE patients, typically exudative, small, and bilateral, associated with peripheral PTE. Although reported in more than one-third of patients with chronic pulmonary embolism, it remains unclear whether acute PTE is associated with reactive hilar and mediastinal lymphadenopathy [28].

In our study, GGO was observed on CTPA in nearly half of patients (48.4%), and parenchymal consolidation in nearly one-third (29.0%); however, neither finding was a significant predictor of PTE severity. These inflammatory findings on CTPA are most likely not directly related to PTE but may result from other pulmonary conditions such as infection, inflammation, or underlying lung disease [44]. Therefore, they cannot independently predict PTE severity.

## 7. Conclusions

Both Daniel’s ECG score and dNLR have been identified as independent, rapid, simple, and cost-effective predictors of PTE severity, available prior to other biochemical or imaging results. Their implementation could substantially enhance early risk stratification of PTE patients through simplified scoring systems applied at emergency admission.

However, given the modest sample size and the single-center design of this study, the generalizability of these findings is limited. Therefore, to validate their predictive value and determine whether Daniel’s score and dNLR improve risk stratification beyond current ESC guidelines, large-scale multicenter randomized controlled trials are necessary.

## 8. Study Limitations

This study has several limitations. It was an observational, single-center study with a limited sample size. Therefore, the results should not be used definitively for clinical decision-making or risk prediction without confirmation from larger, randomized controlled trials. Additionally, due to the small number of high-risk patients, the intermediate-high and high-risk groups were combined; future multicenter studies with larger populations are warranted. Furthermore, patients over 70 years of age were not included, as this study represents a part of a larger research project conducted for a doctoral dissertation.

## Figures and Tables

**Table 1:** The correlation of investigated variables with pulmonary embolism.

Variables	PTE Patients *n* = 69	Control Group *n* = 69	*p*-Value
Sex, n (%)			
-male -female	46 (66.7%) 23 (33.3%)	46 (66.7%) 23 (33.3%)	1.000
Age, mean ± sd	54.9 ± 11.8	55.3 ± 11.6	0.833
Daniel score, median (range)	5 (0–21)	0 (0–11)	<0.001
WBC, mean ± sd	10.5 ± 3.3	7.1 ± 2.1	<0.001
Neutrophils, median (range)	7.5 (1.8–18.2)	3.4 (2.0–9.7)	<0.001
IL 6, median (range)	30.1 (2.7–358.7)	1.5 (1.5–13.3)	<0.001
CRP, median (range)	48.1 (1.6–254.3)	1.3 (0.6–109.0)	<0.001
SII, median (range)	821.1 (208.5–11,946.0)	391.3 (145.4–913.8)	<0.001
NLR score, median (range)	5.3 (1.2–53.5)	1.6 (0.8–3.7)	<0.001
PLR score, median (range)	124.5 (17.5–905.0)	113.3 (44.1–213.5)	0.063
dNLR score, median (range)	3.0 (0.9–21.4)	1.1 (0.62–2.5)	<0.001
NPR score, median (range)	0.038 (0.009–0.378)	0.015 (0.007–0.046)	<0.001
LMR score, median (range)	2.3 (0.3–6.4)	3.7 (2.0–7.3)	<0.001

**Table 2:** PTE location, parenchymal and pleural inflammatory findings on CTPA.

Variables	n = 62	(%)
**PTE location**		
Both main braches	22	35.5
One of the main branches (L or R)	14	22.6
Lobar PTE	18	29.0
Segmental PTE	8	12.9
**Parenchymal and pleural inflammatory findings**		
Ground-glass opacity	30	48.4
Pleural effusion		
-unilateral -bilateral	17 4	27.4 6.5
Consolidation	18	29.0
Mediastinal and hilar lymphadenopathy	19	30.6

**Table 3:** Univariate ordinal logistic regression with degree of PTE as dependent variable.

Variables	Univariate Ordinal Logistic Regression	
	OR (95%CI)	*p*-Value
Sex (Male/Female)	1.10 (0.43–2.78)	0.842
Age	1.04 (0.99–1.08)	0.077
Daniel’s score	1.22 (1.10–1.36)	<0.001
WBC	1.05 (0.92–1.20)	0.458
Neutrophils	1.1 (0.95–1.27)	0.221
IL 6	1.01 (0.99–1.01)	0.182
CRP	1.01 (0.99–1.01)	0.116
SII	1.00012 (0.99989–1.00035)	0.315
NLR	1.08 (1.01–1.16)	0.036
PLR	1.002 (0.999–1.004)	0.258
dNLR	1.25 (1.06–1.48)	0.009
NPR	143.2 (0–4,278,016.4)	0.345
LMR	0.79 (0.55–1.12)	0.179
Ground-glass opacity	2.04 (0.8–5.21)	0.135
Pleural effusions: -unilateral -bilateral	1.96 (0.68–5.66) 1.06 (0.16–7.06)	0.216 0.953
Mediastinal and hilar lymphadenopathy	0.86 (0.27–2.76)	0.806
Consolidation	1.23 (0.44–3.38)	0.695

## Data Availability

The data presented in this study are available on request from the corresponding author due to privacy.
